# Medical Rehabilitation of Patients with Post-COVID-19 Syndrome—A Comparison of Aerobic Interval and Continuous Training

**DOI:** 10.3390/jcm12216739

**Published:** 2023-10-25

**Authors:** Johanna M. Mooren, René Garbsch, Hendrik Schäfer, Mona Kotewitsch, Melina Waranski, Marc Teschler, Boris Schmitz, Frank C. Mooren

**Affiliations:** 1 Department of Rehabilitation Sciences, Faculty of Health, University of Witten/Herdecke, 58455 Witten, Germanyboris.schmitz@uni-wh.de (B.S.); 2 DRV Clinic Königsfeld, Center for Medical Rehabilitation, 58256 Ennepetal, Germany

**Keywords:** SARS-CoV-2, long COVID, aerobic endurance training, severe acute respiratory syndrome, fatigue

## Abstract

Introduction: Post-COVID-19 syndrome (PCS) is a multisystemic disorder marked by impaired physical performance as one lead symptom. Since it has been suggested that endurance training as part of medical rehabilitation may be effective in improving physical performance capacity in PCS, this study aimed to compare different modes of aerobic endurance training. Methods: A total of 110 PCS patients (49.3 ± 11.8 years; 38% women; time after infection = 260.2 ± 127.5 days) underwent detailed clinical screening including symptom-limited cardiopulmonary exercise testing at admission and after 4–6 weeks of inpatient medical rehabilitation. Questionnaires were used to assess disease perception. Patients performed controlled isocaloric cycle ergometer training (3–5 sessions/week; 18 min) as either continuous training (CT) at 50% of maximal workload or as interval training (IT; load = 60%, relief = 30%). Outcomes of PCS patients were compared to coronary artery disease patients (CAD; n = 96) to evaluate overall training effectiveness. Results: Training participation was comparable between the groups, with no indication of training-specific exercise-induced fatigue. Overall, PCS patients improved significantly by a mean of 6.8 ± 12.1% for W at VT1; 3.1 ± 10.0% for VO_2_ at VT1; 5.5 ± 14.7% for O_2_ pulse at VT1; 7.5 ± 15.0% for W at VO_2peak_; 2.7 ± 11.0% for VO_2peak_ and 4.6 ± 12.4% for O_2_ pulse at VO_2peak_ (all *p* < 0.05) with no significant differences between groups (*p* > 0.05). Both groups showed reduced levels of fatigue, anxiety, and depression as well as improved quality of life and wellbeing (all *p* < 0.05). Compared to guideline-based cardiac rehabilitation, PCS patients showed a similar improvement in workload and oxygen uptake compared to CAD patients. Conclusion: PCS patients benefit from aerobic endurance training performed as moderate continuous or interval training as part of a medical rehabilitation program in terms of improved physical exercise capacity and disease perception. The results for PCS patients are comparable to the guideline-based rehabilitation of CAD patients.

## 1. Introduction

Post-COVID-19 syndrome (PCS) occurs as a sequela after acute infection with the SARS-CoV-2 virus (COVID-19 infection). PCS is defined as symptom persistence over a period of 12 weeks from infection and/or the appearance of new symptoms in this period [1]. While PCS characteristics are still a matter of ongoing investigations, recent guidelines identify one of the following criteria to be used for PCS diagnosis [1,2]: symptoms that persist from the acute COVID-19 phase, symptoms that have led to a new health limitation, new symptoms that occurred after the end of the acute phase but are assumed to be a consequence of COVID-19, as well as worsening of a pre-existing underlying disease(s) [1,2]. To some extent, PCS is characterized by diagnostic vagueness, as symptomatology is complex and, due to the lack of diagnostics, not always distinct [3]. In addition, the severity of symptoms is highly variable between patients, ranging from mild impairment to massive restriction of everyday life, including (temporary) partial or complete incapacity to work [2,3]. PCS can be described as a multisystemic disorder; the most common symptoms include (chronic) fatigue, decreased physical performance, muscular weakness and pain, dyspnea, cognitive impairment (memory/brain dysfunction and impaired concentration, also known as brain fog), and mental and psychological distress in the sense of a post-traumatic stress reaction [2,3,4,5]. A COVID-19 infection may trigger various processes that to date are not fully understood, let alone their contribution to PCS. Possible causes involve endothelial dysfunction, a “cytokine storm” associated with increased oxidative stress affecting multiple organs and subcellular structures including mitochondria, central hemodynamics, and others [6]. PCS can occur after severe infection as well as after mild or moderate acute infection, and individual risk factors of PCS are currently controversially discussed [1,7]. Estimates of incidence also vary depending on population and the number/severity of symptoms considered, and the proportion of PCS patients without need for hospitalization during acute infection has been estimated to range from 7.5% to 41% [4,8]. While the majority of affected patients experience a gradual healing process without targeted treatment, the need for effective medical rehabilitation is high at least for patients with persistent PCS [1,4].

Studies aiming to address the efficacy of medical rehabilitation of PCS in general and aerobic endurance training as part of exercise-based rehabilitation in particular are rare, and no guidelines for PCS rehabilitation patients exist. Given that physical exercise has been shown to be beneficial in multiple pathologies such as cardiovascular diseases, neuropathic disorders and pulmonary diseases [9,10], it has been suggested that physical exercise including aerobic training could also exert beneficial effects in PCS [1,11]. However, there is limited evidence on the efficacy of physical exercise in the form of aerobic endurance training to reduce the burden of decreased physical performance in PCS. Moreover, data on the comparison of different training modalities are scarce. Aerobic endurance training is known to positively influence psychological, neurological, cardiovascular, respiratory, and musculoskeletal symptoms in different diseases [12,13], and continuous moderate-intensity training is a 1A guideline recommendation for chronic heart and pulmonary diseases [13,14]. Documented benefits include improvements in various aspects of physiologic function, aerobic exercise capacity, and quality of life [14]. The improvement in aerobic performance is associated with a small-to-moderate increase in peak exercise oxygen uptake, which can already be achieved after several weeks of inpatient or outpatient rehabilitation [11,15]. In recent years, (high-intensity) aerobic interval training has been studied across different cardiovascular-related disorders [15], suggesting equal or even better improvements from interval training compared to continuous training [15,16]. The efficacy of interval training for respiratory and pulmonary functions has been discussed controversially, and no difference between continuous and interval training in patients with chronic obstructive pulmonary disease has been reported [13,17,18]. However, it is crucial to acknowledge the potential detrimental implications of intense physical activity on patients with PCS. Specifically, considering the risk of post-exertional malaise (PEM), too intense interval training may be contraindicated in PCS patients [19]. By contrast, the intermittent nature of interval training could be advantageous for PCS patients by reducing the ventilatory demand associated with exercise, which may also serve to reduce dyspnea and the perceived breathing effort as compared with continuous training [13]. To date, the efficacy of interval training in the medical rehabilitation of PCS has not been reported. Thus, this study aimed to compare moderate-intensity continuous training and interval training for the medical rehabilitation of PCS patients. We hypothesized that both training modalities would result in comparable improvements in terms of overall performance capacity assessed as submaximal and peak oxygen uptake. Finally, the overall trainability of PCS patients was compared to coronary artery disease (CAD) patients undergoing guideline-based medical rehabilitation.

## 2. Materials and Methods

### 2.1. Study Design

**Figure 1:**
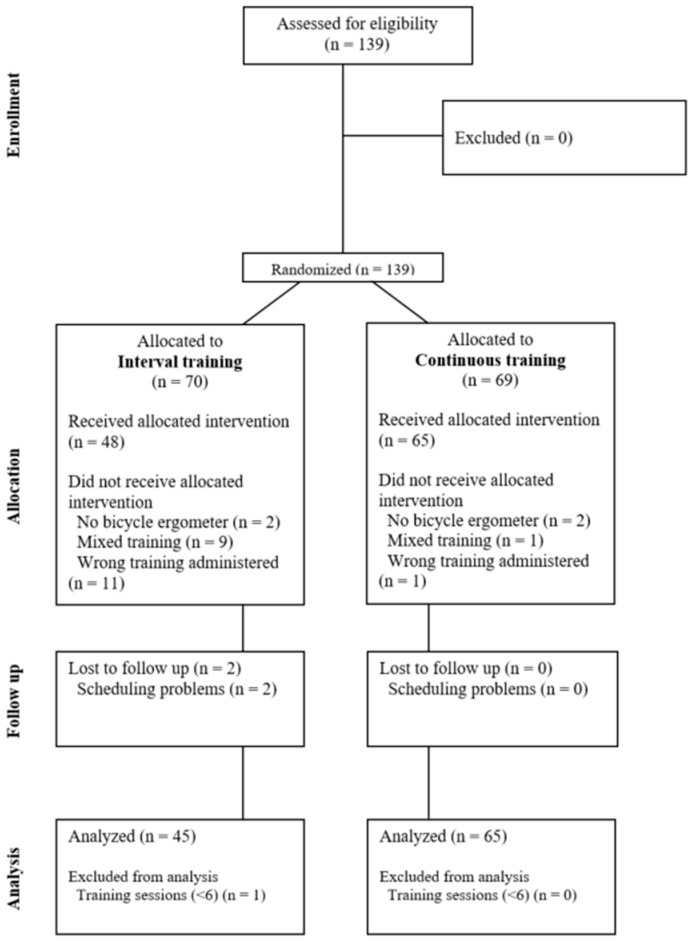
CONSORT Flowchart. In total, 139 patients were randomized into interval training (IT, N = 70) and continuous training (CT, N = 69). For IT, N = 48 and for CT, N = 65 received the allocated intervention. For IT, two patients were lost to follow up due to scheduling problems and one patient was excluded from analysis due to too few training sessions. Finally, an intention-to-treat analysis (ITT) for all (N = 139) and a per-protocol (PP) analysis for 110 (IT, N = 45; CT, N = 65) patients were performed.

A randomized controlled trial (Clinical Trial: NCT06016192) was performed at Clinic Königsfeld between August 2021 and November 2022 including patients with a PCS diagnosis referred for inpatient medical rehabilitation, willingness to participate, and signed informed consent. No further inclusion criteria were defined (Figure 1). After admission, patients were randomized to either continuous training (CT) or interval training (IT) using a computer-generated randomization list. Training was matched for total workload and time (CT = 50% of maximal workload, IT = 60% at load/30% during recovery; total of 18 min), see below for detailed description. A full clinical assessment including symptom-limited cardiopulmonary exercise testing (CPET) was performed at enrollment and before discharge. Validated questionnaires were used to assess changes in disease perception. Patients received individual medical rehabilitation including a combination of strength, respiratory, and cognitive training, as well as physio-, psycho-, and nutrition therapy as indicated. Prescription was unaltered for both groups. PCS patients were compared to patients with CAD undergoing guideline-based medical rehabilitation to estimate the efficacy of physical exercise training. The primary outcome variable was peak oxygen uptake; secondary outcomes included submaximal oxygen uptake (at ventilatory threshold 1 [VT1]) and disease perception.

### 2.2. Study Populations

#### 2.2.1. PCS Patients

In total, 139 PCS patients were randomized and analyzed according to intention to treat (ITT). The per-protocol (PP) analysis included 110 PCS patients. Inclusion criteria were a history of (at least one) COVID-19 infection (positive PCR test at the time of infection), and ongoing or newly expressed performance deficits lasting for at least 3 months prior to recruitment. Performance deficits were documented according to the recent consensus statement, with the cluster of lead symptoms including fatigue/exercise intolerance, shortness of breath, and cognitive dysfunction impairing activity of daily living and everyday functioning [5]. A detailed clinical workup was performed, and the history of comorbidities and current medication was documented. Data on medical rehabilitation including prescriptions of therapeutic actions and participation was recorded.

#### 2.2.2. CAD Patients

A group of 96 patients with a diagnosis of CAD enrolled in a prospective cohort study on the effectiveness of medical rehabilitation was used for comparison. CAD patients after acute myocardial infarction and/or reperfusion via percutaneous transluminal coronary angioplasty were included, matched for age to the enrolled PCS patients. A detailed clinical workup including comorbidities, medication, and cardiopulmonary exercise testing was available for this group. CAD patients performed continuous aerobic exercise training at 50% of maximal workload as part of standardized medical rehabilitation.

#### 2.2.3. Ethical Approval

The study conformed to the Declaration of Helsinki and was approved by the local ethical review committee (Ethik-Kommission Universität Witten/Herdecke; reference number 159/2021 and 115/2020 for PCS and CAD patients, respectively). Written informed consent was obtained from all participants.

#### 2.2.4. Exercise Training

After randomization, bicycle ergometer training (Ergoline Select 100, Ergoline GmbH, Bitz, Germany) was prescribed by clinicians, scheduled by therapy management, and implemented in groups (4–6 patients) by therapists (trained sport scientists and physiotherapists with at least 3 years of professional training). Patients trained 3–5 times per week. Based on preliminary data, the exercise protocol was established at moderate intensities to control fatigue symptomatology and prevent overload by excessive exertion. PCS patients were advised to refrain from training on days of enhanced fatigue. Training in both groups was matched for total workload and time (18 min) as follows. For CT, a standard workload of 50% of maximal workload was applied (identical to guideline-based exercise training in CAD patients). For IT, the workload was 60% at load (100 s) and 30% during recovery (48 s) phases. Adaptation of training was guided by individual training pulse (calculated at the beginning of rehabilitation according to the Karvonen formula) and perceived intensity of the load resulting in adjusted workloads. Both groups trained for a total of 18 min per session with a gradual increase (ramp) at session start until training load was reached. All training sessions were documented by therapists including actual workload and mean heart rate measured by heart rate monitor (chest strap; Ergoline Select 100, Ergoline GmbH). Patients were free to refrain from physical exercise if experiencing side effects but had to be signed off by trainers. In addition, patients received other physical therapies such as (aerobic) group exercise, medical training therapy, aqua fitness, terrain training/walking, and circuit training. Prescription was individualized but identical for both groups. Performed physical therapies (including ergometer training) were documented and converted into metabolic equivalents (METs) according to Ainsworth et al. [20].

#### 2.2.5. Assessment of Perceived Disease Burden, Functional Status, and Fatigue

Disease burden and functional impact on daily life including fatigue was assessed at enrollment and discharge by validated questionnaires. This was also performed to evaluate subjective illness perception in addition to the objective measurement of physical performance. The Multidimensional Fatigue Inventory (MFI-20) was used to assess fatigue [21]. The MFI-20 provides an overall score as well as two subscales on physical and mental fatigue. The scores range from 0–100, with higher scores indicating higher levels of fatigue. Health-related quality of life was assessed using the SF-36 questionnaire, which includes eight health concepts: physical functioning, physical role, bodily pain, general health, vitality, social functioning, emotional role, and mental health. The SF-36 provides two combined scores: a Physical Component Score (PCS) and a Mental Component Score (MCS), ranging between 0 and 100, with higher scores indicating a more favorable functional status [22]. The Hospital Anxiety Depression Scale (HADS) was applied to assess anxiety and depression severities with subscales graded as follows: 0–7 = “normal”, 8–10 = “mild”, 11–14 = “moderate”, and 15–21 = “severe”. The WHO-5 questionnaire was used to evaluate the general level of wellbeing. The score ranges from 0–25; higher scores indicating greater wellbeing [23]. Work ability was measured using the Work Ability Index (WAI) questionnaire only at the time of admission, which includes the following subscales: present working capacity; ability to work concerning the job requirements; diagnosed pathologies; reduction in working capacity due to illness; sick leave over the past 12 months; personal expectations of one’s work skills two years onwards; psychological conditions/resources [24]. The WAI score may be rated low (7–27), moderate (28–36), good (37–43), or excellent (44–49). All questionnaires applied had been validated in German populations [25,26,27,28].

#### 2.2.6. Cardiopulmonary Exercise Testing (CPET)

Symptom-limited ergometer testing with continuous breath-by-breath respiratory gas exchange analysis was performed according to manufacturer’s instructions (Ergostic, Amedtech, Aue, Germany) as part of the general clinical diagnostic routine after admission and within three days before discharge. Expiratory flow measurements were performed by a mass flow sensor, calibrated with a gas mixture of known concentration before each test. Cardiorespiratory fitness of PCS and CAD patients was determined during an initial clinical stress ECG, and an adapted ramp protocol was chosen according to the initial stress ECG result for CPET: 1. low performance (<100 W): start at 20 W, increase by 15 W/2 min; 2. medium performance (100–125 W): start at 20 W, increase by 20 W/2 min; 3. moderate performance (>125 W): start at 25 W, increase 25 W/2 min. Patients were instructed to reach a rating of perceived exertion of ≥8 on the 0–10 Borg Scale during the test. Recorded variables included workload (W), heart rate (HR), oxygen consumption (VO_2_), carbon dioxide production (VCO_2_), and minute ventilation (VE). Peak VO_2_ was defined as the maximal oxygen uptake reported relative to bodyweight and as a percentage of the reference (predicted value corrected for sex, age, and body surface area) for comparability. VO_2_ at the anaerobic threshold (AT; first ventilatory threshold (VT1)) was identified using Ergostic software V.13 and visually confirmed using both the V-slope method and the ventilatory equivalent method (VE/VO_2_). The oxygen pulse was calculated through the VO_2_/HR ratio.

#### 2.2.7. Laboratory Parameters

Blood samples were taken on the day of hospital admittance and were analyzed the same day at an external certified laboratory (accredited for International Organization for Standardization (ISO) 17025 and 15189). In brief, analyses included standard cell populations, HbA1c, C-reactive protein, creatinine, urea, uric acid, lipids, and liver enzymes.

#### 2.2.8. Statistical Analysis

Since no data on interval training in PCS patients are available, power calculation (G*Power, V3.1.9, Germany) was based on the comparison of interval training to moderate endurance training in patients with interstitial lung disease [29] (similar symptoms: dyspnea, fatigue, and low exercise capacity) with an estimated effect size (ES) of 0.6, suggesting a sample size of 120 patients (60 per group, alpha = 0.05, 1-beta = 0.9, per protocol, two-way repeated measures ANOVA). Data were analyzed per protocol (PP) using SPSS (V.28, IBM, Armonk, NY, USA) and GraphPad Prism (V.10, GraphPad Software, Boston, MA, USA). CPET of two PCS patients was not available for analysis because of scheduling problems. Constant variables are expressed as mean ± standard deviation (SD) or median (range) as indicated. Categorical variables are presented as n (%). The non-normal distribution was tested using skewness and kurtosis. Differences between groups over time (CT vs. IT; PCS vs. CAD) were analyzed using a mixed-effects model. The Chi-squared test was used for categorical variables. Differences within group were analyzed using the paired two-sided *t*-test or Wilcoxon test in case of a non-normal distribution. Between-group comparisons (CT vs. IT; PCS vs. CAD) were based on percent-predicted values (percentage of reference, corrected for sex, age, and body surface area) to ensure comparability; within-group comparison was performed using absolute values. Responder analysis was performed for VO_2_ at VT1 and peak exercise as described using the typical error (TE) method and the following equation: $TE={SD}_{\mathrm{diff}}/\sqrt{2}$, where SD_diff_ is calculated as the difference between the variance (SD) of two repeated measures [30]. Responders were defined as participants who demonstrated an increase greater than 2xTE away from zero. Pearson and Spearman rank correlation analyses were performed to investigate correlations between disease perception and physical fitness. Statistical significance was accepted at *p* < 0.05.

## 3. Results

No significant differences between the training groups were detected with respect to anthropometric and clinical data, comorbidities and medication, or perceived disease burden (Table 1 and Table 2). Overall, the mean time interval between the (first) acute COVID-19 infection and the start of medical rehabilitation was 260.2 ± 127.5 days with no significant differences between groups (IT, 253.4 days ± 117.2 days; CT, 264.9 ± 134.8 days; *p* = 0.646). During acute infection, 28.2% of PCS patients had been hospitalized (IT, 33.3%; CT, 24.6%; *p* = 0.390) and 71.8% of PCS patients had received ambulant care or acute care at home (IT, 66.7%; CT, 75.4%). The overall mean length of inpatient rehabilitation was 28.8 ± 6.1 days with no significant difference between groups (IT, 27.9 ± 5.4 days; CT, 29.4 ± 6.4 days; *p* = 0.202). Overall, 39.1% were never smokers (IT, 40%; CT, 38.5%; ns).

### 3.1. Disease Perception

To assess patients’ perceived impairment of physical performance, disease severity, and disease impact on patients’ daily life, different standardized questionnaires were used (Table 2). The results indicated an overall high level of both physical and mental fatigue (MFI-20 score, 69.8 ± 13.2) at admission. The overall workability was low (22.1 ± 7.4) with a median maximum incapacity for work during the last 12 months of 99 days. The SF-36 questionnaire indicated a low health-related quality of life in the physical (30.4 ± 7.8) and mental (35.7 ± 12.2) component score at baseline. In addition, baseline wellbeing, anxiety, and depression showed significant deviations from normal. Of note, correlation analyses revealed a significant association of the SF-36 physical component score and maximal exercise capacity assessed as peak oxygen uptake (T0, *p* = 0.027, R = 0.222; T1, *p* = 0.005, R = 0.297) as well as workability index and peak oxygen uptake (T0, *p* = 0.025, R = 0.234). No correlation of exercise capacity was detected with the other questionnaires used. As a response to the exercise-based medical rehabilitation, patients improved in all relevant domains from T0 to T1, including the subcategories of physical and mental components, without any significant differences between the groups (Table 2).

### 3.2. Baseline Performance and Physical Exercise Training

At baseline, PCS patients’ performance in peak oxygen uptake was restricted to 18.3 ± 4.4 mL/min/kg resulting in 74.4 ± 15.3% of predicted reference, not differing between groups (peak oxygen uptake: IT, 18.5 ± 4.3 mL/min/kg; CT, 18.2 ± 4.5 mL/min/kg) (Table 3). The analysis of performed ergometer sessions revealed that the actual initial workload (load phase) was slightly lower at the prescribed intensity, while both interventions were isocaloric (net load: IT 47.2 ± 5.4%; CT 47.4 ± 6.5% of max. workload). Overall, PCS patients participated in 92.7 ± 10.7% of prescribed ergometer training sessions (IT, 89.5 ± 11.3%; CT, 94.9 ± 9.6%; *p* = 0.012). The difference in participation between the groups did not correlate with fatigue or PEM, as no indications of training-specific exercise-induced fatigue or PEM were detected (Table 4). During the intervention, patients’ overall training workload was increased by 12.5 ± 12.4 W, with no significant difference between groups (IT, 11.0 ± 11.8 W; CT, 13.5 ± 13.7 W; *p* = 0.307), while the target HR remained unaltered. Of note, participation in additional physical therapies did not differ between the groups, and ther total METs achieved during rehabilitation were comparable (IT 105.8 ± 30.9 METs; CT 107.3 ± 33.0 METs; *p* = 0.833).

### 3.3. Effect of Training Modalities on Physical Exercise Capacity

**Figure 2:**
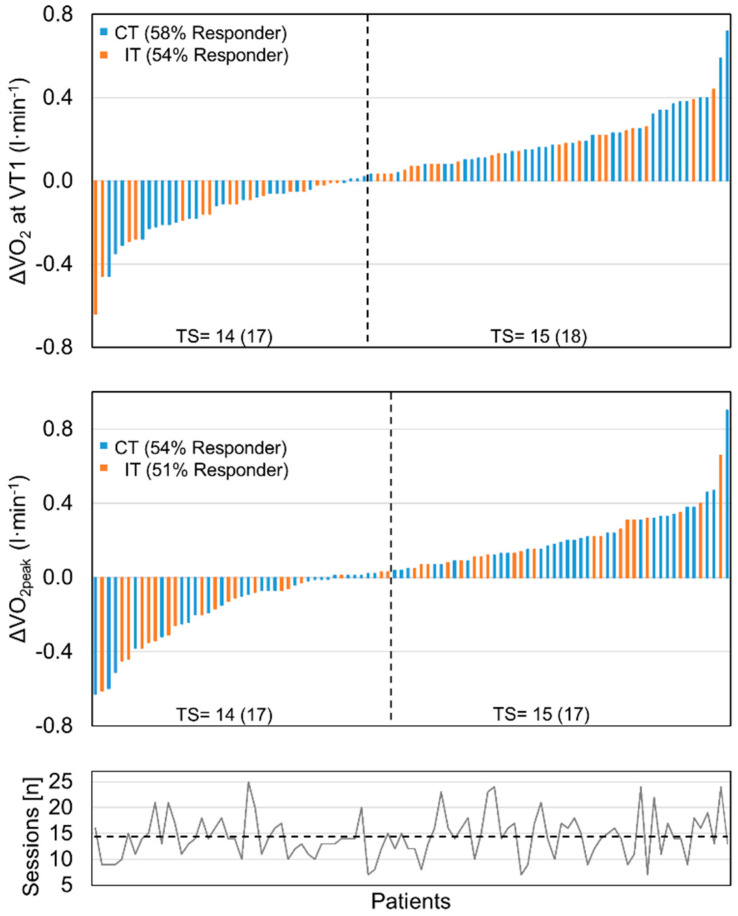
Training response of PCS patients (N = 95) was comparable between continuous training (CT) and interval training (IT). Above: responder analysis based on change in VO_2_. Individual changes of patients are presented as delta between baseline (T0) and discharge (T1) at ventilatory threshold 1 (VT1) and VO_2peak_ in ascending order. Vertical line separates non-responder (left) and responder (right). Continuous training (CT) is indicated in blue; interval training (IT) is indicated in orange. Median number of training sessions (TS) is given below. The typical error (TE) method (2xTE away from zero) was used to define responders as described [30]. Below: overall and individual training sessions performed. Horizontal dashed line indicates the median of overall training sessions (TS) performed (14 (range 7–25)); individual training sessions are indicated by grey line.

The per-protocol analysis revealed no differences between the two training modalities (Table 3) in physical exercise capacity at submaximal (VT1) and peak load (VO_2peak_). Peak oxygen uptake increased by 0.2 ± 2.9 mL/min/kg for IT and 1.3 ± 2.9 mL/min/kg for CT, resulting in an improvement of 0.8 ± 11.2% for IT and 4.1 ± 10.8% for CT, with no significant difference between the groups (*p* = 0.157). Submaximal oxygen uptake at VT1 increased by 0.3 ± 2.3 mL/min/kg for IT and 1.2 ± 2.7 mL/min/kg for CT with no difference between the training modalities. Of note, the application of stringent criteria for maximal exercise testing (RER ≥ 0.95 and Borg ≥ 8) revealed comparable results. The responder analysis suggested that both groups responded equally to training at submaximal load (IT, 56%; CT, 58%; *p* = 0.835) and at peak exercise (IT, 51%; CT, 54%; *p* = 0.836) (Figure 2). An overall analysis of training response with respect to sex, age, or mean number of performed training sessions did not suggest any effect of these variables. An intention-to-treat (ITT) analysis with imputation of missing values using linear regression [31] (five models) yielded similar results.

### 3.4. Comparison of Medical Rehabilitation Effectiveness on Physical Performance Improvement

**Figure 3:**
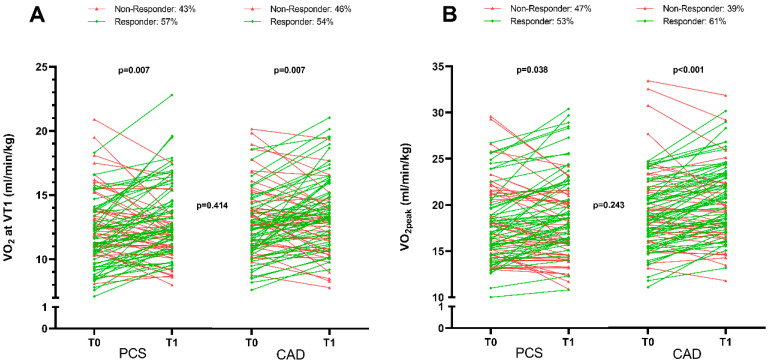
Comparison of exercise training results between PCS (N = 95) and CAD patients (N = 93). PCS and CAD patients showed a comparable response to physical exercise training in medical rehabilitation. A comparison of individual slopes (baseline (T0) to discharge, (T1)) of relative oxygen uptake of PCS patients (n = 96, 40% women) and CAD patients (n = 96, 25% women) is shown for (**A**) submaximal (VT1) and (**B**) peak (VO_2peak_) exercise intensity. PCS patients increased their oxygen uptake significantly at submaximal and maximal intensities, with a comparable increase in exercise capacity seen for PCS patients and CAD patients. Non-responder (red) and responder (green) were defined using the typical error (TE) method (2xTE away from zero) [30]. Within-group comparison from T0 to T1 was performed using absolute oxygen uptake and paired two-sided *t*-test. Between-group comparison was performed using percent-predicted values (reference values) corrected for sex, age, and body surface area by unpaired two-sided *t*-test.

To rate the observed overall improvements of physical exercise capacity in PCS patients at the submaximal and peak oxygen uptake of 2.7 ± 11.0% and 3.1 ± 10.0% (*p* ≤ 0.022) (Table 3), respectively, these findings were compared to the guideline-based rehabilitation of CAD patients (Supplemental material, Table S1). In general, the mean length of inpatient rehabilitation of CAD patients was 24.2 ± 3.7 days, during which a median of 12 training sessions were performed. In terms of exercise capacity at baseline, PCS as well as CAD patients started at a comparably low level (Supplemental material, Table S2), recorded by peak workload compared to the reference (PCS, 73.4 ± 21.1%; CAD, 74.4 ± 21.3%; *p* = 0.739) and peak oxygen uptake (PCS, 74.4 ± 15.3%; CAD, 74.5 ± 15.7%; *p* = 0.982). The improvement in performance between the two groups was comparable in terms of peak oxygen uptake (ΔVO_2peak_: PCS, 2.7 ± 11.0%; CAD, 3.2 ± 12.1%; *p* = 0.796). The responder analysis revealed that both groups responded equally to training in terms of submaximal and peak exercise capacity (Figure 3). Of note, PCS patients showed a significantly higher improvement in workload at VT1 (ΔW at VT1: PCS, 10.5 ± 20.1 W (6.8 ± 12.1%); CAD, 3.8 ± 20.0 W (2.1 ± 8.6%); *p* = 0.011).

## 4. Discussion

This study primarily compared the efficacy of moderate aerobic interval and continuous exercise training for the improvement of the physical exercise capacity of patients with long-term post-COVID-19 syndrome (PCS) during inpatient medical rehabilitation. Furthermore, the performance outcomes of PCS patients were compared to CAD patients performing guideline-based medical rehabilitation. In brief, the key findings of this study are that (1) moderate-intensity aerobic continuous and interval training are equally effective in improving physical exercise capacity of PCS patients during medical rehabilitation, (2) regular physical exercise at moderate intensity can improve submaximal and peak exercise capacity in long-term PCS patients, and (3) moderate-intensity exercise training as part of medical rehabilitation in PCS patients ameliorates performance deficits to a comparable extent, as in CAD patients undergoing guideline-based rehabilitation.

PCS is a multifaceted clinical condition that is characterized by reduced physical and cognitive performance and includes lead symptoms such as fatigue, shortness of breath, and cognitive dysfunction, as well as moderate to severe reductions in physical capacity [5]. The pathophysiological mechanisms underlying the PCS-specific systemic performance decrease are a matter of ongoing investigations and may involve alterations in various tissues and functions. Alterations in hemostasiology and the microvasculature structure leading to impaired oxygen transfer at different locations across the alveolo-capillary membrane and the erythrocyte membrane, as well as entry into muscle cells, have been discussed [6,32]. Likewise, a reduction in peak oxygen uptake (VO_2peak_) during cardiopulmonary exercise testing has been observed in our cohort and others [33,34]. It has thus been suggested that moderate-intensity aerobic endurance training may improve physical performance deficits in PCS patients during rehabilitation, and exercise performance improvements were reported after an 8-week outpatient rehabilitation program [11] with three training sessions per week (30 to 60 min, total of 24 sessions) at moderate intensity (80% of lactate threshold), leading to an improvement of 2.7 mL/min/kg peak oxygen uptake. These results were reported by an early study involving 50 patients after a known COVID-19 infection (between March and November 2020) three months after hospital discharge who presented with ≤85% of predicted peak oxygen uptake (VO_2peak_). While the majority (~70%) of PCS patients in our series had not been hospitalized during acute infection, patients were referred to medical rehabilitation for symptom persistence or new symptom onset after a mean time of ~260 days presenting with high levels of fatigue and largely reduced workability. The RECOVE trial (rehabilitation for post-COVID-19 condition through a supervised exercise intervention) confirmed these results in non-hospitalized PCS patients treated with tailored multicomponent exercise training for 8 weeks (three supervised sessions per week) resulting in an improvement of 2.1 mL/min/kg in maximal oxygen consumption. Also, this study showed a significantly higher quality of life, less fatigue, less depression, and improved functional status compared to controls [35]. In comparison, the patients in our study who presented with a slightly higher level of cardiopulmonary exercise capacity at admission showed a somewhat smaller improvement in maximal oxygen uptake of 0.8 mL/min/kg, which might be explained by the different inclusion criteria and the ~40% shorter treatment period. However, both studies provide evidence that regular aerobic physical exercise training as part of medical rehabilitation can improve exercise capacity in PCS patients with mild to moderate symptom severeness, while Ostrowska et al. (2023) showed increased physical capacity determined by the 6-min walking test, 30 s chair stand test, and a short physical performance battery, which were not reflected in peak oxygen uptake [36]. The comparison of moderate aerobic continuous training to moderate-intensity interval training did not suggest differences in training-induced improvements of exercise capacity. Of note, patients in both groups were able to increase their training load to a comparable extent, and no pattern of training-specific exercise-induced fatigue was observed. For the interval training group with a higher workload during the loading phase, this might also be based on the relatively short load phase followed by a respective recovery phase as well as the related lower breathing effort. Future studies are needed to investigate whether interval training with higher workload or a longer duration during load phases may be tolerated by PCS patients to further increase the improvement in exercise capacity. In terms of perceived impairment of physical performance, disease severity, and disease impact on patients’ daily life, both groups showed equal benefits from medical rehabilitation, resulting in less fatigue and depression as well as improved quality of life and wellbeing, which is in line with findings from the RECOVE trial [35].

Since no guidelines for medical rehabilitation for PCS exist, and no consensus for physical exercise training has been reached, we compared the observed improvements in PCS patients to CAD patients participating in guideline-based inpatient medical rehabilitation [37] involving submaximal endurance training with gradual increase. Our data provide evidence that PCS patients benefit to a similar extent from aerobic endurance training during medical rehabilitation in terms of improved cardiopulmonary exercise capacity based on a comparison corrected for sex, age, and body surface area using percent-predicted values. Overall, our data indicate that PCS patients comparable to those enrolled in our study are trainable, and rehabilitation may lead to improvements in exercise capacity. However, at discharge, PCS and CAD patients were at ~75% of peak exercise capacity, presenting a need for an efficient maintenance program to further improve physical exercise capacity in both patient groups. Endurance training is known to cause numerous adaptations at different functional levels, relating to the cardiopulmonary system, energy and lipid metabolism, as well as mitochondrial, circulatory, and hematologic adaptations [38]. Comparable training effects in CAD and PCS patients suggest that PCS patients are able to adapt to aerobic endurance training despite the multisystemic nature of the disease and potentially persistent multi-organ dysfunction, with prolonged cardiovascular derangements also occurring during recovery [6].

Some limitations to the present study may exist. Even though no significant differences between the two training groups existed, the skewed group sizes and implementation error may have affected the comparability of both groups. However, the comparison of the main outcome variables was performed using percent-predicted values corrected for sex, age, and body surface area. Differences in medical rehabilitation programs between PCS and CAD patients exist due to the nature of the disease, which limits the comparability to some extent. Finally, even though PCS patients enrolled in this study were characterized by long-term symptom persistence, they were capable of participating in a medical rehabilitation program, and our findings may not be transferred to PCS patients with greater symptom severity.

## 5. Conclusions

In summary, we provide evidence that PCS patients benefit from aerobic endurance training, performed as continuous or interval training as part of a medical rehabilitation program, in terms of improved physical exercise capacity and perceived disease burden. Training-specific exercise-induced fatigue was not detected, suggesting that both training modalities were tolerable and effective for PCS patients and may be used interchangeably considering patients’ preferences. In comparison to guideline-based medical rehabilitation in CAD patients, the performance gains of PCS patients appeared to be equal, suggesting a general trainability in the specific patient group analyzed in this study. Since fatigue is a common symptom of PCS, which may interfere with patients’ ability to participate in exercise and limit the training response, an assessment of fatigue and respective pacing strategies may be used to further improve the effectiveness of aerobic endurance training in medical rehabilitation.

## Figures and Tables

**Table 1:** Anthropometric and clinical data, medication and blood parameters.

	Overall (n = 110)	IT (n = 45)	CT (n = 65)	*p*-Value
**Anthropometric data**				
**Age, years**	49.3 ± 11.8	50.5 ± 10.1	48.4 ± 12.8	0.359
**Sex, n (%)**				0.693
female	42 (38.2)	16 (35.6)	26 (40.0)	
male	68 (61.8)	29 (64.4)	39 (60.0)	
**Height, cm**	174.6 ± 9.5	174.5 ± 9.2	174.7 ± 9.7	0.924
**Weight, kg**	92.5 ± 21.0	93.3 ± 18.2	91.9 ± 22.8	0.372
**BMI, kg** **·m^−2^**	30.2 ± 6.0	30.6 ± 5.7	29.9 ± 6.2	0.270
**Clinical data**				
**Diseases of the circulatory system, n (%)**	72 (65.6)	29 (64.4)	43 (66.2)	0.855
Arterial hypertension	55 (50)	22 (48.9)	33 (50.8)	0.848
Pulmonary embolism	10 (9.1)	4 (8.9)	6 (9.2)	0.952
Paroxysmal tachycardia	15 (13.6)	4 (8.9)	11 (16.9)	0.231
Coronary artery disease	3 (2.7)	2 (4.4)	1 (1.5)	0.405
Other	23 (20.9)	8 (17.8)	15 (23.1)	0.506
**Endocrine, nutritional, or metabolic diseases, n (%)**	73 (66.4)	31 (68.9)	42 (64.6)	0.645
Obesity	57 (51.8)	26 (57.8)	31 (47.7)	0.302
Type 2 diabetes mellitus	12 (10.9)	3 (6.7)	9 (13.8)	0.239
Hypothyroidism, n (%)	11 (10.0)	3 (6.7)	8 (12.3)	0.337
Other	25 (22.7)	8 (17.8)	17 (26.2)	0.307
**Diseases of the musculoskeletal system and connective tissue, n (%)**	43 (39.1)	12 (26.7)	31 (47.7)	0.026
**Diseases of the nervous system, n (%)**	28 (25.5)	13 (28.9)	15 (23.1)	0.502
Migraine/headache	3 (2.7)	1 (2.2)	2 (3.1)	0.789
Other	25 (22.7)	12 (26.7)	13 (20.0)	0.426
**Mental and behavioral disorders, n (%)**	28 (25.5)	13 (28.9)	15 (23.1)	0.502
Depressive/adjustment disorders	19 (17.3)	9 (20.0)	10 (15.4)	0.542
Other	10 (9.1)	4 (8.9)	6 (9.2)	0.952
**Diseases of the respiratory system, n (%)**	15 (13.6)	8 (17.8)	7 (10.8)	0.316
**Diseases of the digestive system, n (%)**	14 (12.7)	5 (11.1)	9 (13.8)	0.676
**Neoplasms, n (%)**	7 (6.4)	2 (4.4)	5 (7.7)	0.497
**Medication**				
**ACE inhibitor**	19 (17.3)	8 (17.8)	11 (16.9)	0.909
**Statin**	20 (18.2)	8 (17.8)	12 (18.5)	0.928
**Beta blocker**	40 (36.4)	16 (35.6)	24 (36.9)	0.885
**AT-II receptor blocker**	24 (21.8)	11 (24.4)	13 (20.0)	0.588
**Calcium channel blocker**	21 (19.1)	11 (24.4)	10 (15.4)	0.254
**Anticoagulant**	22 (20.0)	8 (17.8)	14 (21.5)	0.632
**Antiarrhythmic**	1 (0.9)	0 (0)	1 (1.5)	0.408
**Diuretic**	22 (20.0)	12 (26.7)	10 (15.4)	0.165
**Glucocorticoid**	18 (16.4)	7 (15.6)	11 (16.9)	0.851
**Analgesic**	26 (23.6)	10 (22.2)	16 (24.6)	0.774
**Antidepressant**	15 (13.6)	7 (15.6)	8 (12.3)	0.636
**Diabetes medication**	6 (5.5)	1 (2.2)	5 (7.7)	0.218
**Blood Parameters**				
**Leukocytes, n/nL**	6.9 ± 1.8	7.0 ± 1.8	6.8 ± 1.9	0.674
**Erythrocytes, M/µL**	4.9 ± 0.4	4.9 ± 0.4	4.9 ± 0.5	0.847
Red cell distribution width (RDW), %	13.1 ± 0.9	13.1 ± 0.7	13.2 ± 1.1	0.568
Hemoglobin, g/dL	14.5 ± 1.4	14.7 ± 1.2	14.4 ± 1.5	0.314
Hematocrit, %	43.6 ± 3.8	44.0 ± 3.3	43.3 ± 4.1	0.368
Mean corpuscular volume (MCV), fl	90.0 ± 5.3	90.6 ± 3.2	89.7 ± 6.3	0.407
Mean corpuscular hemoglobin (MCH), pg	30.0 ± 2.1	30.2 ± 1.1	29.8 ± 2.5	0.330
Mean cellular hemoglobin concentration (MCHC), g/dL	33.3 ± 1.1	33.4 ± 0.8	33.2 ± 1.2	0.474
**Thrombocytes, n/nL**	254.6 ± 67.1	261.1 ± 54.0	250.2 ± 74.8	0.402
**HbA1c, %**	6.1 ± 0.8	6.1 ± 0.7	6.0 ± 0.9	0.891
**C-reactive protein, mg/dL**	0.4 ± 0.6	0.3 ± 0.3	0.4 ± 0.7	0.424
**Sodium, mmol/L**	141.0 ± 2.1	141.5 ± 2.2	140.6 ± 2.1	0.023
**Potassium, mmol/L**	4.3 ± 0.4	4.3 ± 0.4	4.3 ± 0.4	0.401
**Creatinine, mg/dL**	0.9 ± 0.2	0.9 ± 0.2	0.8 ± 0.2	0.438
**Urea, mg/dL**	30.5 ± 8.0	30.4 ± 7.2	30.6 ± 8.5	0.882
**eGFR, mL/min/1.73qm**	95.5 ± 15.0	93.7 ± 15.0	96.8 ± 15.1	0.305
**Uric acid, mg/dL**	5.9 ± 1.5	6.1 ± 1.4	5.7 ± 1.5	0.100
**Triglycerides, mg/dL**	166.7 ± 112.0	169.0 ± 89.6	165.1 ± 125.8	0.858
**Cholesterol, mg/dL**	207.5 ± 49.4	207.6 ± 60.0	207.5 ± 41.0	0.989
**HDL cholesterol, mg/dL**	55.1 ± 16.7	55.3 ± 17.8	55.1 ± 16.0	0.947
**LDL cholesterol, mg/dL**	134.0 ± 43.5	136.5 ± 52.7	132.2 ± 36.1	0.635
**LDL/HDL ratio**	2.6 ± 1.0	2.6 ± 0.9	2.6 ± 1.1	0.890
**Creatin kinase, U/L**	159.2 ± 125.7	163.2 ± 110.6	156.6 ± 135.7	0.789
**Glutamate oxalacetate transaminase (GOT), U/L**	32.6 ± 13.1	34.2 ± 15.7	31.6 ± 12.0	0.322
**Glutamate pyruvate transaminase (GPT), U/L**	39.7 ± 23.1	42.1 ± 19.9	38.0 ± 25.1	0.370
**Gamma glutamyl transferase (gamma GT), U/L**	38.9 ± 26.5	45.0 ± 32.8	34.8 ± 20.5	0.071
**Alkaline phosphatase, U/L**	79.4 ± 25.5	77.8 ± 23.1	80.4 ± 27.1	0.599
**Thyroid stimulating hormone (TSH), mlU/L**	1.4 ± 0.8	1.5 ± 0.9	1.2 ± 0.6	0.164

Data are presented as mean ± SD or n (%). Between-group comparison was performed using unpaired two-sided *t*-test, Mann–Whitney U test, or Chi-squared test. Diseases/symptoms with a prevalence <5% are not reported. IT, interval training; CT, continuous training; BMI, body mass index. eGFR was calculated using CKD-EPI formula.

**Table 2:** Perceived disease severity.

	Overall	IT	CT	*p*-Value
	(n = 110)	(n = 45)	(n = 65)	
**Multidimensional fatigue inventory (MFI-20)**				
Overall Score				
T0	69.8 ± 13.2	68.2 ± 14.9	70.9 ± 11.9	0.339
T1	58.6 ± 15.9	54.3 ± 14.6	61.4 ± 16.1	0.029 ^#^
Δ	−11.3 ± 15.0 ***	−14.2 ± 12.6	−9.4 ± 16.3	0.124
Physical Fatigue				
T0	77.2 ± 15.5	76.3 ± 16.8	77.8 ± 14.1	0.663
T1	62.8 ± 19.2	57.7 ± 17.7	66.1 ± 19.6	0.033 ^#^
Δ	−14.3 ± 20.5 ***	−19.1 ± 18.9	−11.3 ± 21.0	0.062
Mental Fatigue				
T0	65.5 ± 20.0	64.3 ± 18.9	66.3 ± 20.8	0.621
T1	57.0 ± 19.9	53.1 ± 18.8	59.6 ± 20.2	0.112
Δ	−9.1 ± 16.2 ***	−11.7 ± 14.9	−7.5 ± 16.9	0.212
**SF-36 Health-related quality of life**				
Physical Component Score (PCS)				
T0	30.4 ± 7.8	31.3 ± 7.0	29.9 ± 8.3	0.366
T1	35.2 ± 9.1	38.0 ± 8.8	33.4 ± 8.9	0.015 ^#^
Δ	4.6 ± 7.2 ***	6.9 ± 7.3	3.1 ± 6.7	0.013 ^#^
Mental Component Score (MCS)				
T0	35.7 ± 12.2	36.5 ± 11.2	35.1 ± 12.9	0.573
T1	41.7 ± 11.5	41.5 ± 11.6	41.9 ± 11.6	0.852
Δ	6.0 ± 10.2 ***	6.0 ± 8.5	6.0 ± 11.2	0.984
**Wellbeing (WHO-5)**				
T0	8 (22)	8 (20)	8 (18)	0.356
T1	14 (24)	15 (23)	12.5 (22)	0.126
Δ	4 (26) ***	5 (22)	4 (26)	0.301
**Hospital anxiety and depression scale (HADS)**				
Anxiety				
T0	7 (16)	8 (15)	6.5 (16)	0.19
T1	6 (18)	6 (18)	5.5 (15)	0.935
Δ	0 (18) **	0 (17)	0 (18)	0.628
Depression				
T0	7 (19)	8 (16)	7 (19)	0.64
T1	5 (20)	5 (20)	5 (20)	0.681
Δ	0 (22) *	0 (22)	0 (17)	0.71
**Workability Index (WAI) ^$^**	22.1 ± 7.4	23.7 ± 8.0	21.1 ± 6.8	0.104
max. incapacity for work last 12 months ^§^	99 (365)	99 (365)	99 (365)	0.859

Perceived disease severity was assessed by questionnaire at baseline and before discharge. Data are presented as mean ± SD or median (range). Between-group comparison was performed using mixed-effects model. Within-group comparison for overall data was performed using paired two-sided *t*-test or Wilcoxon test. MFI-20: range 0–100 (higher = greater fatigue); WAI: range 7–49 (higher = improved work ability); SF-36: range 0–100 (higher = greater quality of life); WHO-5: range 0–25 (higher = greater wellbeing); HADS: range 0–21 (higher = greater anxiety/depression). ^$^ WAI was only assessed at admission, as items refer to the period of the last 12 months. ^§^ Patient-reported days being off work because of illness (WAI item; the maximal number of days was used to calculate the group mean). IT, interval training; CT, continuous training. * *p* < 0.05, ** *p* < 0.01, *** *p* < 0.001, significantly different from T0 to T1 (all *p* ≤ 0.043). ^#^ Significantly different between groups.

**Table 3:** Changes in exercise capacity assessed by cardiopulmonary exercise test (CPET).

	Overall	IT	CT	*p*-Value
	(n = 110)	(n = 45)	(n = 65)	
**Resting**				
**Heart rate, beat·min^−1^**				
T0	89.1 ± 11.5	88.3 ± 11.2	89.7 ± 11.7	0.545
T1	83.3 ± 11.6	83.6 ± 11.1	83.1 ± 12.0	0.82
Δ	−6.0 ± 10.1 ***	−4.0 ± 9.7	−7.2 ± 10.2	0.126
**O_2_ pulse, mL·beat^−1^**				
T0	6.8 ± 1.9	6.8 ± 1.8	6.8 ± 1.9	0.915
T1	6.8 ± 1.6	7.0 ± 1.4	6.6 ± 1.6	0.169
Δ	−0.0 ± 1.5	0.2 ± 1.4	−0.2 ± 1.5	0.313
**Ventilatory equivalent O_2_ (VE/VO_2_)**				
T0	28.0 ± 5.9	27.0 ± 6.3	28.5 ± 5.6	0.21
T1	28.2 ± 6.9	27.2 ± 4.9	29.0 ± 7.9	0.218
Δ	0.2 ± 7.3	0.2 ± 6.7	0.2 ± 7.7	0.982
**Ventilatory equivalent CO_2_ (VE/VCO_2_)**				
T0	33.9 ± 8.8	33.8 ± 12.5	34.1 ± 5.0	0.88
T1	35.1 ± 9.7	33.4 ± 3.8	36.2 ± 12.0	0.162
Δ	1.0 ± 12.6	−0.5 ± 13.3	1.9 ± 12.2	0.367
**Ventilatory threshold 1 (VT1)**				
**Workload, W (% predicted)**				
T0	73.7 ± 27.1 (42.5 ± 14.9)	73.5 ± 26.9 (42.3 ± 14.2)	73.8 ± 27.4 (42.7 ± 15.5)	0.887
T1	85.1 ± 25.0 (50.2 ± 15.7)	84.9 ± 22.3 (50.6 ± 14.1)	85.2 ± 27.0 (49.9 ± 16.9)	0.821
Δ	10.5 ± 20.1 (6.8 ± 12.1) ***	10.4 ± 22.5 (6.8 ± 12.9)	10.6 ± 18.3 (6.8 ± 11.5)	0.998
**Heart rate, beat·min^−1^ (% predicted)**				
T0	109.3 ± 13.7 (65.4 ± 7.7)	107.4 ± 11.9 (64.7 ± 7.3)	110.6 ± 14.7 (65.9 ± 7.9)	0.429
T1	106.6 ± 20.3 (64.2 ± 11.3)	105.5 ± 14.5 (64.3 ± 8.4)	107.3 ± 23.7 (64.1 ± 13.1)	0.945
Δ	−3.8 ± 16.9 (−2.3 ± 10.6)	−1.5 ± 12.7 (−1.0 ± 7.7)	−5.4 ± 19.3 (−3.2 ± 12.2)	0.318
**O_2_ pulse, mL·beat^−1^ (% predicted)**				
T0	10.6 ± 2.9 (77.2 ± 16.2)	11.0 ± 2.8 (79.7 ± 17.3)	10.3 ± 2.9 (75.5 ± 15.2)	0.197
T1	11.3 ± 2.8 (83.5 ± 17.1)	11.5 ± 2.4 (85.3 ± 16.0)	11.2 ± 3.1 (82.2 ± 17.9)	0.381
Δ	0.7 ± 1.8 (5.5 ± 14.7) ***	0.4 ± 1.3 (3.2 ± 8.9)	1.0 ± 2.1 (7.2 ± 17.7)	0.196
**VO_2_, mL·min^−1^·kg^−1^ (% predicted)**				
T0	12.4 ± 3.0 (50.5 ± 11.7)	12.5 ± 2.9 (51.4 ± 12.1)	12.4 ± 3.1 (50.0 ± 11.5)	0.534
T1	13.2 ± 3.4 (54.6 ± 11.7)	12.8 ± 2.9 (54.7 ± 11.9)	13.5 ± 3.7 (54.6 ± 11.6)	0.941
Δ	0.8 ± 2.5 (3.1 ± 10.0) *	0.3 ± 2.3 (1.4 ± 8.9)	1.2 ± 2.7 (4.3 ± 10.6)	0.164
**Ventilatory equivalent O_2_ (VE/VO_2_) (% predicted)**				
T0	27.4 ± 4.7 (78.3 ± 13.4)	27.1 ± 3.8 (77.3 ± 10.9)	27.6 ± 5.2 (79.0 ± 14.9)	0.541
T1	26.6 ± 3.9 (75.9 ± 11.2)	26.7 ± 4.1 (76.3 ± 11.7)	26.5 ± 3.8 (75.7 ± 10.9)	0.771
Δ	−0.8 ± 4.4 (−2.1 ± 12.5)	−0.4 ± 3.5 (−1.0 ± 10.1)	−1.0 ± 4.9 (−2.9 ± 14.0)	0.486
**Ventilatory equivalent CO_2_ (VE/VCO_2_)**				
T0	30.6 ± 4.4	30.0 ± 3.4	31.0 ± 4.9	0.223
T1	29.6 ± 3.8	29.8 ± 3.6	29.5 ± 3.9	0.687
Δ	−0.9 ± 3.3 *	−0.3 ± 2.6	−1.3 ± 3.7	0.121
**Peak exercise**				
**Respiratory exchange rate (RER) (% predicted)**				
T0	1.05 ± 0.1 (87.1 ± 7.7)	1.06 ± 0.1 (87.3 ± 6.7)	1.05 ± 0.1 (87.0 ± 8.3)	0.815
T1	1.05 ± 0.1 (87.1 ± 6.4)	1.06 ± 0.1 (87.4 ± 6.2)	1.05 ± 0.1 (86.8 ± 6.6)	0.634
Δ	0.00 ± 0.1 (0.2 ± 6.3)	0.00 ± 0.1 (0.0 ± 5.4)	−0.0 ± 0.1 (−0.4 ± 6.8)	0.758
**Workload, W (% predicted)**				
T0	127.8 ± 38.5 (73.4 ± 21.1)	130.2 ± 38.0 (74.1 ± 20.7)	126.2 ± 39.0 (72.9 ± 21.5)	0.775
T1	140.1 ± 39.2 (81.0 ± 24.6)	142.5 ± 37.1 (84.2 ± 19.5)	138.3 ± 40.9 (78.7 ± 27.8)	0.283
Δ	12.6 ± 18.3 (7.5 ± 15.0) ***	11.6 ± 18.5 (8.7 ± 14.7)	13.4 ± 18.4 (6.6 ± 15.3)	0.507
**Heart rate, beat·min^−1^ (% predicted)**				
T0	135.3 ± 21.2 (80.9 ± 11.5)	134.2 ± 18.3 (80.7 ± 10.4)	136.1 ± 23.0 (81.0 ± 12.3)	0.89
T1	132.2 ± 20.2 (79.6 ± 10.9)	129.7 ± 17.0 (79.0 ± 9.4)	134.0 ± 22.2 (80.1 ± 11.9)	0.645
Δ	−2.6 ± 13.9 (−1.4 ± 8.5)	−3.3 ± 12.4 (−1.9 ± 7.6)	−2.2 ± 15.1 (−1.1 ± 9.1)	0.642
**O_2_ pulse, ml·beat^−1^ (% predicted)**				
T0	12.7 ± 3.3 (92.6 ± 17.4)	13.1 ± 3.3 (94.5 ± 18.4)	12.4 ± 3.2 (91.4 ± 16.7)	0.37
T1	13.3 ± 3.0 (97.9 ± 16.0)	13.6 ± 2.8 (100.1 ± 17.2)	13.1 ± 3.1 (96.3 ± 15.0)	0.266
Δ	0.6 ± 1.8 (4.6 ± 12.4) ***	0.3 ± 1.6 (2.8 ± 11.5)	0.8 ± 1.9 (5.9 ± 12.9)	0.233
**VO_2_, mL·min^−1^·kg^−1^ (% predicted)**				
T0	18.3 ± 4.4 (74.4 ± 15.3)	18.5 ± 4.3 (75.7 ± 14.8)	18.2 ± 4.5 (73.6 ± 15.7)	0.482
T1	18.9 ± 4.8 (77.8 ± 15.6)	18.7 ± 4.4 (78.8 ± 14.8)	19.1 ± 5.2 (77.1 ± 16.3)	0.592
Δ	0.8 ± 2.9 (2.7 ± 11.0) *	0.2 ± 2.9 (0.8 ± 11.2)	1.3 ± 2.9 (4.1 ± 10.8)	0.157
**Ventilatory equivalent O_2_ (VE/VO_2_) (% predicted)**				
T0	33.8 ± 6.1 (96.5 ± 17.5)	33.6 ± 5.5 (96.0 ± 15.7)	33.9 ± 6.6 (96.8 ± 18.7)	0.826
T1	33.1 ± 5.1 (94.4 ± 14.5)	33.3 ± 5.8 (95.1 ± 16.5)	32.9 ± 4.5 (93.9 ± 12.9)	0.699
Δ	−0.6 ± 4.6 (−1.7 ± 13.1)	−0.3 ± 3.3 (−1.0 ± 9.5)	−0.8 ± 5.4 (−2.2 ± 15.3)	0.658
**Ventilatory equivalent CO_2_ (VE/VCO_2_)**				
T0	32.1 ± 5.1	31.8 ± 4.3	32.3 ± 5.7	0.601
T1	31.4 ± 4.3	31.4 ± 4.5	31.4 ± 4.1	0.942
Δ	−0.5 ± 3.6	−0.4 ± 2.5	−0.6 ± 4.2	0.733
**Respiratory minute ventilation (VE), L·min^−1^ (% predicted)**				
T0	60.3 ± 15.0 (58.0 ± 13.3)	61.3 ± 14.1 (58.4 ± 12.2)	59.6 ± 15.7 (57.8 ± 14.1)	0.807
T1	60.9 ± 15.8 (59.0 ± 13.4)	61.5 ± 14.8 (59.7 ± 13.0)	60.4 ± 16.7 (58.4 ± 13.8)	0.643
Δ	0.9 ± 11.0 (1.3 ± 10.6)	−0.3 ± 10.4 (0.2 ± 9.7)	1.7 ± 11.4 (2.0 ± 11.2)	0.417
**Tidal volume (Vt), L (% predicted)**				
T0	2.1 ± 0.6 (72.4 ± 23.6)	2.1 ± 0.5 (73.1 ± 21.1)	2.1 ± 0.6 (71.9 ± 25.3)	0.792
T1	2.2 ± 0.6 (74.4 ± 20.6)	2.2 ± 0.6 (74.1 ± 19.2)	2.2 ± 0.6 (74.7 ± 21.8)	0.887
Δ	0.1 ± 0.3 (2.1 ± 23.3)	0.1 ± 0.3 (1.6 ± 21.9)	0.1 ± 0.3 (2.4 ± 24.4)	0.882
**Breathing frequency (Bf), breaths·min^−1^ (% predicted)**				
T0	30.1 ± 8.0 (54.7 ± 14.5)	29.9 ± 6.2 (54.4 ± 11.3)	30.2 ± 9.1 (54.9 ± 16.4)	0.874
T1	29.1 ± 6.8 (53.0 ± 12.4)	29.4 ± 6.4 (53.5 ± 11.5)	28.9 ± 7.2 (52.6 ± 13.0)	0.729
Δ	−0.6 ± 4.9 (−1.1 ± 8.8)	−1.1 ± 4.1 (−2.0 ± 7.2)	−0.3 ± 5.4 (−0.4 ± 9.7)	0.386
**Breathing reserve (BR), %**				
T0	37.9 ± 19.5	36.1 ± 19.7	39.1 ± 19.4	0.44
T1	37.6 ± 18.0	37.2 ± 18.0	37.9 ± 18.1	0.853
Δ	0.6 ± 15.9	3.5 ± 13.9	−1.5 ± 17.0	0.129

Data are presented as mean ± SD at T0 (baseline) and T1 (discharge) with respective changes (delta). If indicated, percent of predicted values adjusted for sex, age, and body surface area are provided. Between-group comparisons were performed using mixed-effects model with predicted values, if applicable. Within-group comparison for overall data was performed using paired two-sided *t*-test or Wilcoxon test with absolute values. IT, interval training; CT, continuous training. * *p* < 0.05, *** *p* < 0.001, significantly different from T0 to T1 (all *p* ≤ 0.046).

**Table 4:** Exercise training data.

	Overall (n = 110)	IT (n = 45)	CT (n = 65)	*p*-Value
**Sessions prescribed, n**	15 (20)	16 (16)	14 (20)	0.125
**Sessions performed, %**	92.7 ± 10.7	89.5 ± 11.3	94.9 ± 9.6	0.012
**Workload Increase, %**	10.1 ± 10.5	9.6 ± 10.1	10.4 ± 10.9	0.695
**Exercise heart rate, beat·min^−1^**	112.2 ± 14.8	113.9 ± 14.4	111.1 ± 15.0	0.317

Data are presented as mean ± SD, median (range) or n (%). Between-group comparison was performed using unpaired two-sided *t*-test or Mann–Whitney U test. Workload increase indicates change in net workload from first to last training session. Exercise heart rate was calculated as mean over all performed training sessions. IT, interval training; CT, continuous training.

## Data Availability

The datasets used in this study are available from the corresponding author upon reasonable request.

## Supplementary Materials

The following supporting information can be downloaded at: https://www.mdpi.com/article/10.3390/jcm12216739/s1, Table S1. Anthropometric and clinical data of CAD patients. Table S2. Changes in exercise capacity of CAD patients.
