# Supplementary material for: Medical Rehabilitation of Patients with Post-COVID-19 Syndrome—A Comparison of Aerobic Interval and Continuous Training
Source: J Clin Med. 2023 Oct 25;12(21):6739. doi: 10.3390/jcm12216739 (PMC10649998; doi:10.3390/jcm12216739)
Supplement: Supplementary file 1 [file jcm-12-06739-s001.zip › jcm-2624095-supplementary.pdf]

Supplementary Material

Table S1. Anthropometric and clinical data of CAD patients.

	CAD (n=96)
**Anthropometric data**	
**Age, years**	50.3 ± 6.6
**Sex, n (%)**	
female	24 (25.0)
male	72 (75.0)
**Height, cm**	174.6 ± 8.5
**Weight, kg**	92.2 ± 17.3
**BMI, kg*m^{-2}**	30.1 ± 4.8
**Clinical data**	
**Coronary artery disease, n (%)**	96 (100)
One vessel disease	43 (44.8)
Two vessel disease	32 (33.3)
Three vessel disease	21 (21.9)
**Myocardial infarction, n (%)**	83 (86.5)
STEMI	52 (54.2)
NSTEMI	31 (32.3)

Only main diagnoses are listed. Data is presented as mean ± SD or n (%). BMI, Body Mass Index; STEMI, ST-Elevation Myocardial Infarction; NSTEMI, Non-ST-Elevation Myocardial Infarction.

Table S2. Changes in exercise capacity of CAD patients.

	CAD (n=96)
**Ventilatory threshold 1 (VT1)**	
**Workload, W (% predicted)**	
T0	73.6 ± 25.9 (42.5 ± 13.7)
T1	78.8 ± 28.0 (44.2 ± 13.7)
Δ	3.8 ± 20.0 (2.1 ± 8.6)***
**VO_2, ml·min^{-1}·kg^{-1} (% predicted)**	
T0	12.6 ± 2.8 (59.3 ± 21.0)
T1	13.3 ± 2.9 (64.2 ± 24.6)
Δ	0.7 ± 2.3 (4.6 ± 20.0)**
**Peak exercise**	
**Workload, W (% predicted)**	
T0	128.0 ± 41.9 (74.4 ± 21.3)
T1	137.6 ± 44.6 (79.4 ± 22.2)
Δ	9.5 ± 26.4 (5.0 ± 18.0)***
**VO_2, ml·min^{-1}·kg^{-1} (% predicted)**	
T0	19.1 ± 4.4 (74.5 ± 15.7)
T1	20.1 ± 4.6 (77.6 ± 16.4)
Δ	1.0 ± 2.8 (3.2 ± 12.1)**

Data is presented as mean ± SD, at T0 (baseline), T1 (discharge) with respective changes (delta). Absolut values and percent of predicted values adjusted for sex, age and body surface area are provided. Within-group comparison was performed using paired two-sided t-test or Wilcoxon-Test based on absolute values. CAD, coronary artery disease patients. *p<0.05, **p<0.01, ***p<0.001.
